# Desiccation tolerance in *Anopheles coluzzii*: the effects of spiracle size and cuticular hydrocarbons

**DOI:** 10.1242/jeb.135665

**Published:** 2016-06-01

**Authors:** Arthur C. Arcaz, Diana L. Huestis, Adama Dao, Alpha S. Yaro, Moussa Diallo, John Andersen, Gary J. Blomquist, Tovi Lehmann

**Affiliations:** 1 Laboratory of Malaria and Vector Research, NIAID, NIH, Rockville, MD 20852, USA; 2 Malaria Research and Training Center (MRTC)/Faculty of Medicine, Pharmacy and Odonto-stomatology, Bamako, BP 1805, Mali; 3 Department of Biochemistry and Molecular Biology, University of Nevada, Reno, NV 89557, USA

**Keywords:** Mosquito, Aestivation, *Anopheles gambiae*, Desiccation resistance, Water balance, Dry season

## Abstract

The African malaria mosquitoes *Anopheles gambiae* and *Anopheles coluzzii* range over forests and arid areas, where they withstand dry spells and months-long dry seasons, suggesting variation in their desiccation tolerance. We subjected a laboratory colony (G3) and wild Sahelian mosquitoes during the rainy and dry seasons to desiccation assays. The thoracic spiracles and amount and composition of cuticular hydrocarbons (CHCs) of individual mosquitoes were measured to determine the effects of these traits on desiccation tolerance. The relative humidity of the assay, body water available, rate of water loss and water content at death accounted for 88% of the variation in desiccation tolerance. Spiracle size did not affect the rate of water loss or desiccation tolerance of the colony mosquitoes, as was the case for the total CHCs. However, six CHCs accounted for 71% of the variation in desiccation tolerance and three accounted for 72% of the variation in the rate of water loss. Wild *A. coluzzii* exhibited elevated desiccation tolerance during the dry season. During that time, relative thorax and spiracle sizes were smaller than during the rainy season. A smaller spiracle size appeared to increase *A. coluzzii*'s desiccation tolerance, but was not statistically significant. Seasonal changes in CHC composition were detected in Sahelian *A. coluzzii*. Stepwise regression models suggested the effect of particular CHCs on desiccation tolerance. In conclusion, the combination of particular CHCs along with the total amount of CHCs is a primary mechanism conferring desiccation tolerance in *A. coluzzii*, while variation in spiracle size might be a secondary mechanism.

## Abstract (precis)

**Summary:** Seasonal variation in desiccation tolerance of malaria mosquitoes is shaped by cuticular hydrocarbon composition and quantity and possibly by smaller spiracles. Seasonal allometries indicate season-specific developmental programs, consistent with aestivation.

## INTRODUCTION

The importance of *Anopheles gambiae* and *Anopheles* *coluzzii* (previously the S and M forms of *A. gambiae*, respectively) in the transmission of malaria is widely recognized (WHO, 2014), yet despite extensive research, major gaps remain in our understanding of fundamental aspects of their biology. The ranges of these vectors cover steamy forests, dry savannas and semi-arid areas in Africa, contributing greatly to their role in disease transmission. These sibling species withstand dry conditions during short dry spells in the rainy season (RS) as well as the 3–8 month-long dry season (DS). Their presence in both arid and humid environments suggests variability in their desiccation tolerance (DT), as was previously hypothesized (Coluzzi, 1982; Coluzzi et al., 1979; Toure et al., 1994, 1998). Recent evidence suggests that during the DS, *A. coluzzii* persists in the Sahel via a form of dormancy (aestivation) whereas *A. gambiae* engages in long-distance migration to re-colonize Sahelian villages in the RS (Dao et al., 2014; Huestis and Lehmann, 2014; Lehmann et al., 2010). Eco-physiological studies revealed surprisingly modest variation in DT between species and carriers of chromosomal inversions that were associated with aridity clines geographically and seasonally (Fouet et al., 2012; Gray et al., 2009; Lee et al., 2009; Lyons et al., 2014) – findings that are difficult to reconcile with the distinct environments they inhabit. The mechanisms underlying DT in anopheline mosquitoes are poorly understood. Such knowledge might help uncover subpopulations with elevated or reduced DT associated with aestivation or other ecophysiological states.

Adult anophelines have two pairs of spiracles in the thorax and seven considerably smaller pairs in the abdomen (Snodgrass, 1959). We focused on the large metathoracic and mesothoracic spiracles, which vary in length (Nagpal et al., 2003; Wagoner et al., 2014), yet this variation has not been examined in terms of its effect on DT. Mosquitoes close their spiracles presumably to optimize the balance between gas exchange and water loss (Gray and Bradley, 2006) based on external and internal factors, such as relative humidity (RH), hydration and starvation status (Chown and Nicolson, 2004; Krafsur, 1971). In most insects, a muscle contraction closes the spiracles, which open because of the elasticity of the thoracic cuticle (Miller, 1960). During flight, all four thoracic spiracles are open, presumably to maximize gas exchange (Herreid and Fourtner, 1981). In *Drosophila*, at least one spiracle is partially open even in extremely dry environments to allow the organism to breathe (Gibbs et al., 2003).

The primary role of the insect epicuticle is to minimize evaporation, although it also protects against radiation, infection and injury (Wigglesworth, 1933; Hadley, 1994). The waxy epicuticle is composed mainly of hydrocarbons and other lipids (Hadley, 1994; Haverty et al., 1991; Howard and Blomquist, 2005; Page et al., 1990). An increased amount of total cuticular hydrocarbons (CHCs) as well as changes in CHC composition were linked to elevated DT in insects (Bazinet et al., 2010; Benoit and Denlinger, 2007; Chown and Nicolson, 2004; Chung and Carroll, 2015; Denlinger and Armbruster, 2014; Gibbs et al., 1997; Gibbs and Rajpurohit, 2010; Howard and Blomquist, 2005). The rate of water loss (RWL) of insects increases rapidly above a ‘transition’ temperature – an observation that led to the phase-transition model that describes cuticle permeability as a function of temperature-dependent melting of its surface lipids (Wigglesworth, 1945). Although not all evidence is consistent with the model's predictions, most biophysical and eco-physiological studies showed that populations and species from warmer and drier environments tend to exhibit more lipids that presumably melt at higher temperatures (Gibbs and Pomonis, 1995; Gibbs and Rajpurohit, 2010). In *A. gambiae s.l.*, 48 CHCs were identified, ranging in chain length from 17 to 47 carbons (Caputo et al., 2007, 2005). Variation in CHC composition in anopheline mosquitoes has been linked to species (Caputo et al., 2007; Milligan et al., 1993), age (Caputo et al., 2005; Hugo et al., 2006) and mating status (Polerstock et al., 2002). The possibility that CHC composition plays a dual role in mate recognition and hence in speciation, while at the same time affecting tolerance to arid conditions (Chung and Carroll, 2015), is of special interest given the contentious speciation processes in this species complex (Coetzee et al., 2013; Coluzzi et al., 1985; della Torre et al., 2002; Lehmann and Diabate, 2008; Simard et al., 2009). However, as in the case of spiracle size, the effect of variation in CHC composition on DT, although hypothesized, has not been empirically evaluated in mosquitoes to the best of our knowledge.

Abbreviations

BWA: body water available, defined as wet mass−end mass (mg)
BWCD: body water content at death, defined as (end mass−dry mass)/end mass (%)
CHC: cuticular hydrocarbon
DS: dry season
DT: desiccation tolerance, expressed as survival time (h)
RH: relative humidity (%)
RS: rainy season
RWL: rate of water loss, defined as BWA/DT, i.e. the loss of body water available over desiccation time (mg h^−1^)
WL: wing length measured from the alular notch to the junction of the radius 3 vein and the outer margin (mm)

Water loss in insects occurs by evaporation across the cuticle, by transpiration through spiracles and via excrement (Gray and Bradley, 2005; Hadley, 1994; Toolson and Hadley, 1987). Here, we investigated the mechanisms underlying DT in laboratory and field *A. coluzzii* mosquitoes, focusing on the roles of the size of the spiracles and of the CHCs. We tested the hypotheses that compared with mosquitoes collected during the RS, those collected in the DS exhibit (i) greater DT, (ii) a smaller relative spiracle size and (iii) a higher total amount of CHCs and changes in their composition. Further, we hypothesized that the changes in CHC composition will include (iv) a reduction in the relative quantity of alkenes, methyl-branched and short-chain alkanes that would result in elevating the melting temperature of CHCs and thus decrease permeability of the cuticle to water, in accordance with the phase transitional model (Gibbs and Rajpurohit, 2010). Finally, we hypothesized that (v) similar mechanisms in Sahelian *A. coluzzii* also operate in a related laboratory colony. Although the order of these predictions follows our conceptual scheme, the laboratory experiment offers greater control over key parameters and it is treated earlier in the Results section.

## MATERIALS AND METHODS

### Laboratory DT experiment

The *A. gambiae* G3 colony is maintained in an insectary (27°C, 80% RH, 12 h:12 h light:dark photoperiod) as described previously (Artis et al., 2014). The RH of the two treatment chambers was set at 20% and 80%, simulating the conditions in the Sahelian DS and RS, respectively (Huestis and Lehmann, 2014). The humidity in each treatment was maintained using supersaturated salt solutions of potassium fluoride (KF) and potassium chloride (KCl; Sigma, St Louis, MO, USA), respectively. The salt solutions had a sludge-like consistency when poured into Petri dishes to maximize the surface area of each solution. Five Petri dishes were placed in separate plastic, air-tight containers (50×40×20.3 cm). Humidity and temperature were monitored in the containers using HOBO data-loggers (Onset Computer Corporation, Bourne, MA, USA). In the dry and the humid treatment chambers, mean (±s.e.m.) RH and temperature were 22.0±0.11% RH (min.=20.1%, max.=43.1%; *N*=978) and 27.6±0.005°C (min.=26.5°C, max.=27.9°C; *N*=978) and 80.7±0.019% RH (min.=77.6%, max.=82.4%; *N*=984) and 27.7±0.005°C (min.=26.6°C, max.=28.2°C; *N*=978), respectively. Each treatment was replicated in two identical air-tight containers.

One hundred, 3 day old *A. gambiae* G3 females were individually weighed using a microbalance (AD 6000, PerkinElmer, Waltham, MA, USA) to the nearest 1 µg, after light anesthesia in diethyl ether (Mallinckrodt Chemicals, St Louis, MO, USA) for 6 s. This initial mass is referred to as ‘wet mass’. Mosquitoes were then placed individually into a 50 ml centrifuge tube covered with a net, and then randomly assigned to one of the two treatments, described above. Individuals were placed in containers after the RH reached within 5% of the expected value. The treatment chambers were set inside a controlled insectary chamber maintained at 28°C with a 13 h:11 h light:dark photoperiod, without access to food or water.

After 1 h, and thereafter every 2 h, mosquitoes were checked for mortality, defined by their inability to stand upright when the tubes were agitated. Upon verification of death, mosquitoes were immediately weighed to obtain their ‘end mass’, transferred to a closed 1.5 ml tube with desiccant (Sigma) and heated to 65°C in a desiccating oven (HB-1D, Techne, Staffordshire, UK). After 4 days in the desiccating oven, the samples were re-weighed to obtain their ‘dry mass’.

The mosquitoes in each treatment were ranked based on survival duration under the desiccating assay. The top and bottom 20%, as well as 10 randomly selected mosquitoes from the center of the distribution (for a total of 50 mosquitoes), were dissected by removing the wings and legs in order to obtain morphological measurements and subsequently CHC measurements.

### Field DT assay

#### Study sites and mosquito collection

Indoor resting mosquitoes were collected (using mouth aspirators) between 08:00 h and 10:00 h in February and August 2011 from the village of Thierola, Mali (13.6583N, 7.2155W), located in the Sahel. The last rains fall in October and no surface water can be found over a 30 km radius from December to the end of May (Dao et al., 2014; Lehmann et al., 2010). The climate in the area has been described previously (Huestis and Lehmann, 2014). Collected mosquitoes were identified visually as *A. gambiae s.l.* and classified by sex and gonotrophic state. They were kept in the shade, covered with a wet towel until subjected to the desiccation assay around 11:00 h the same day.

Mosquitoes were placed individually into modified 15 ml tubes that were cut 7 cm from the top, and the open side was covered by a net (the top was kept on). These inserts were placed in 50 ml tubes with 15 g of Drierite™, a calcium sulfate desiccant (W. A. Hammond Drierite Company, Ltd, Xenia, OH, USA), with the netted opening pointing up (to avoid contact between the mosquito and the desiccant). Parafilm was wrapped around the top of the 50 ml tube as an additional sealant. Samples were checked for mortality every 2 h (between 00:00 h and 06:00 h, inspection was done every 3 h). Upon death (defined as above), mosquitoes were individually placed into a 1.5 ml vial with silica gel and kept in direct sunlight for 12 h to expedite desiccation. To identify the species and molecular form, PCR and restriction enzyme assays were conducted on two legs (Fanello et al., 2002). The specimens were shipped to the NIH (MD, USA) for additional measurements (below).

### Morphological measurements

Body size was estimated by wing length (WL). Thirteen wing landmarks were used to measure WL of *A. gambiae* G3 mosquitoes (Huestis et al., 2011). The same distance was measured on *A. coluzzii* using a dissecting microscope equipped with micrometer ruler under ×40 magnification to the nearest 0.1 mm (Yaro et al., 2006). After the wings and legs had been removed, mosquitoes were placed on a slide with their lateral side facing up. All morphological landmarks were taken using a 3D digital microscope (Starlite 200, Quality Vision International, Rochester, NY, USA) using a routine that was created in Measure-X software (RAM Optical, Rochester, NY, USA). Both left and right lateral sides were measured for each mosquito and averaged (unless damage on one side prevented landmark identification, in which case only one side was used). Thorax size was estimated based on three landmarks on the thorax (Fig. S1). A combination of eight and 13 points around the contour of the metathoracic spiracles and mesothoracic spiracles, respectively, were used to assess the size of each spiracle (Fig. S2). The length, perimeter and area were calculated using functions in Measure-X.

### CHC measurement

CHCs were extracted and analyzed by gas chromatography (GC). Each sample was submerged in 12 μl of solvent (10 ppm pentadecane standard in hexane) for 10 min. A 2 μl sample of the extraction solution was injected into an Agilent 6850 Series II gas chromatograph (Agilent Technologies, Santa Clara, CA, USA) equipped with a HD-1 column (30 m, 0.32 mm, 0.25 μm) heated from 75 to 150°C at 25°C min^−1^ and then from 150 to 310°C at 8°C min^−1^ and then held at 310°C for an additional 10 min. The injector was set at 300°C. Helium was used as the carrier gas (constant flow of 1 ml min^−1^) and the injection mode was set on pulsed splitless [25 psi (∼172 kPa) pulse pressure]. A 20 Hz data collection rate was used. EZChrom SI data system (Agilent Technologies, Santa Clara, CA, USA) was used to calculate the area under the peaks (standard option).

Output of the gas chromatograph was visually inspected and occasional corrections of peak area were manually made using split peak and alignment of the base of each peak. Exceptionally broad peaks with height to area ratio below 0.12 (5.9% of the total peaks) were excluded. Bins were constructed based on the clusters of the peaks’ retention times and the gaps between clusters. Bins were identified by matching their retention time with that of standard alkane mix (C_21_-C_40_, Sigma-Aldrich 04071), which was diluted 1:9 with our pentadecane standard solution) and run under the same GC program. Peaks were putatively identified as specific CHCs based on alignment with these standards and with peaks previously identified by GC-MS using a pool of 30 G3 females of mixed gonotrophic state and ranging in age from 2 to 15 days (and separately, a pool of 30 G3 males, both mated and virgin). Other peaks were putatively categorized as oxygenated lipids (mostly fatty acids) and excluded from the analysis.

### Data analysis

Mosquito DT was estimated as midpoint survival based on the last time the mosquito was observed alive and the time it was found dead. Mosquitoes that died in the first 2 h were excluded from the DT analyses because of the concern that their death was due to handling accidents.

For the laboratory experiment, body water available (BWA) was defined as wet mass−end mass (mg), whereas body water content at death (BWCD) was defined as (end mass−dry mass)/end mass (%). The RWL was defined as BWA/DT (mg h^−1^) and expressed the loss of body water available over the desiccation time. Because the microbalance for measuring the mass of individual mosquitoes was not available in the field, only DT was measured in the field experiment. The effect size of independent variables in multivariate ANOVA was estimated using ‘semipartial’ ω^2^ statistics, i.e. after all other effects were partialed out of the effect in question (without adjustment on the dependent variable). This measures the adjusted effect as a proportion of the dependent variation remaining after partialing all other effects. This adjustment, based on the covariance with all other factors in the model may result in a lower value than the corresponding *r*^2^ values in a univariate regression model.

Prior to binning of GC-trace peaks, adjustments were made to peak time and peak area based on the time and area of the pentadecane standard. Peaks with standardized area below 1% of the pentadecane and singleton peaks were excluded. The amount of total CHCs of each specimen was then estimated as the sum of the (adjusted) areas of all CHCs. The CHC composition was expressed as the proportion of each (adjusted) peak area of the total (defined above). The resulting compositional data were transformed to minimize interdependence among peaks by the log contrast transformation (Aitchison, 1986), using *n*-C29 (time=22.7 min) as the common denominator for each mosquito, i.e. transformed-CHn=log(proportion CHn/proportion *n*-C29). The denominator peak must be present in all specimens, but its identity has no effect on the results (Aitchison, 1986).

The absence of a particular CHC peak from certain specimens complicated the analysis of compositional data because of the uncertainty of whether the absence reflected a detection problem or a true absence (Aitchison, 1986). We analyzed both the fraction of specimens that exhibited each CHC as well as the relative quantity of each CHC after imputation of a small value (−3.5) when the log contrast transform value was not defined due to zero. This value was selected because the transformed value of the lowest detected CHC peak was −3.45. Imputation was not performed for CHCs that were observed in <4 specimens in both seasons (or treatments in the laboratory experiment). To address multiple tests, we used the binomial test (which estimates the probability of obtaining the observed number of significant tests at the 0.05 level given the total number of tests) as a global test to detect departure from the null hypothesis, such as across multiple CHCs, and the sequential Bonferroni test (Holm, 1979) to detect whether a particular significant test on the individual test level is significant given the total tests performed. Statistical analyses were performed using SAS 9.3 (SAS, 2011).

## RESULTS

### Laboratory experiment

#### DT and body water content

**Fig. 1.**
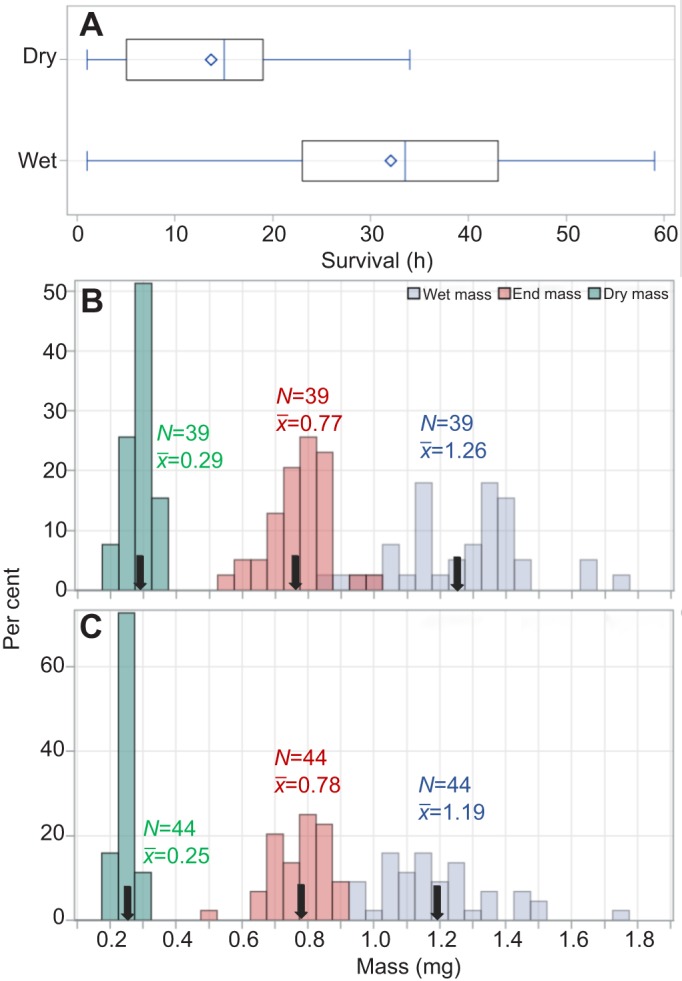
**Variation in desiccation tolerance and changes in body mass values in 3** **day old G3 colony mosquitoes under dry and humid treatments.** (A) Variation between treatments in desiccation tolerance (DT), measured as survival time (*N*_dry_=39 and *N*_wet_=44). The mean and median survival time are denoted by a diamond and a line (dividing the box), respectively. (B) Changes in body mass distribution before the assay (wet mass) and at the end of the assay (end mass), and dry mass under the dry treatment (20% relative humidity, RH). Arrows (black) point to the mean mass (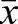, shown above the arrow with sample size) of each group. (C) Same as in B for the humid treatment (80% RH).

Mosquitoes subjected to the desiccation assay survived longer in the high humidity treatment: 36.2 versus 16.3 h (*P*<0.0001, ANOVA, *F*_1,95_=47.8). Considerable variation in survival (DT) within treatment was indicated by the spread of the values (Fig. 1A). On average, mosquitoes lost 36% of their initial wet mass during the assay, corresponding to a reduction of ∼13% in body water content (from 78% to 65%). There was no significant difference in initial (wet) mass and in the end mass between treatments (*P*>0.062, *F*_1,81_>3.56, ANOVA; Fig. 1B,C); however, the dry mass of the mosquitoes subjected to the dry treatment was higher than that of the mosquitoes subjected to the humid treatment (*P*<0.001, *F*_1,81_>40.8, ANOVA; Fig. 1B,C), indicating that a small part of the mass lost during the assays was likely nutritional reserves rather than water.

**Fig. 2.**
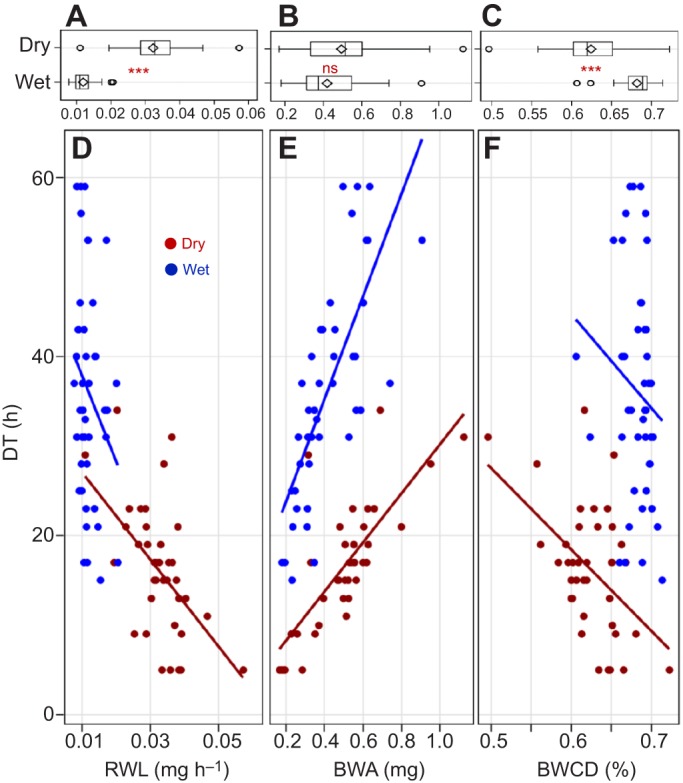
**Variability in the components of water balance and their interrelationships.** Variation in the rate of water loss (RWL; A), body water available (BWA; B) and body water content at death (BWCD; C), and their relationship to DT (D–F) in the G3 colony by treatment (*N*_dry_=39, *N*_wet_=44).

The RWL increased at lower RH (0.032 versus 0.012 mg h^−1^, *P*<0.0001, *F*_1,81_>221.4, Welch's ANOVA; Fig. 2A), and so did its variance (*P*<0.0003, *F*_1,81_>15.2, Brown and Forsythe's test; Fig. 2A), with few mosquitoes under dry conditions exhibiting RWL values as low as those under high RH. The total amount of water lost (BWA) during the assay (0.49 and 0.42 mg in the dry and humid treatments, respectively) was not different between treatments (*P*>0.06, *F*_1,81_>3.5, Welch's ANOVA; Fig. 2B), yet BWCD was reduced under dry conditions (0.64% versus 0.68%, *P*<0.0001, *F*_1,81_>69.4, Welch's ANOVA; Fig. 2C), and its variance was larger (*P*<0.004, *F*_1,81_>8.9, Brown and Forsythe's test; Fig. 2C), suggesting that the capacity to withstand a greater water loss is enhanced under dry conditions. However, this might reflect the higher dry mass of mosquitoes subjected to the dry treatment (above). The relationships of these components of mosquito water balance and DT were evaluated graphically (Fig. 2D–F) and their specific effects evaluated in a multivariate regression model. After removal of insignificant interaction terms, the model accounted for 88% of the variation in DT. The component-specific effect sizes, after partialing out correlation with other factors (see Materials and methods), revealed that the amount of water available for a mosquito explains most of the variation in DT (25%) followed by RWL (15%) and treatment (5%; Table 1). Note that in this formulation, the ambient RH effect is mostly subsumed in RWL. BWCD accounted for only 1% and its confidence interval included zero. As expected, increasing RWL or BWCD decreased DT, whereas larger amounts of initial body water, increased DT (Table 1).

**Table 1.**
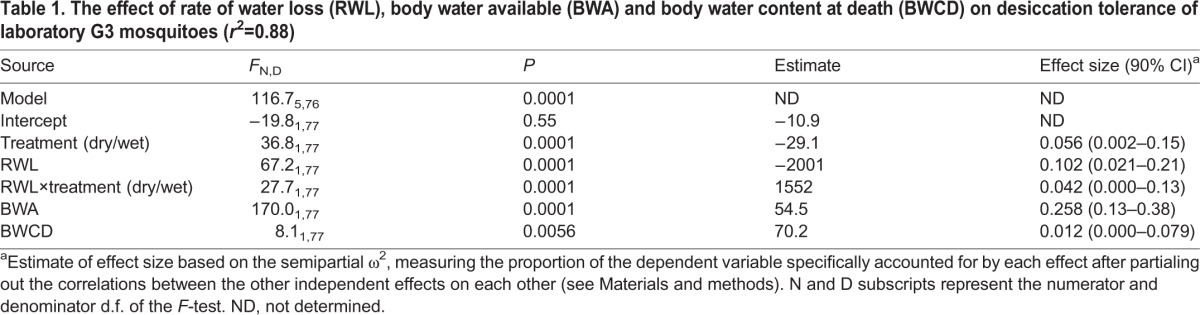
**The effect of rate of water loss (RWL), body water available (BWA) and body water content at death (BWCD) on desiccation tolerance of laboratory G3 mosquitoes (*r*^2^=0.88)**

#### Body size and spiracle size effects on DT

**Fig. 3.**
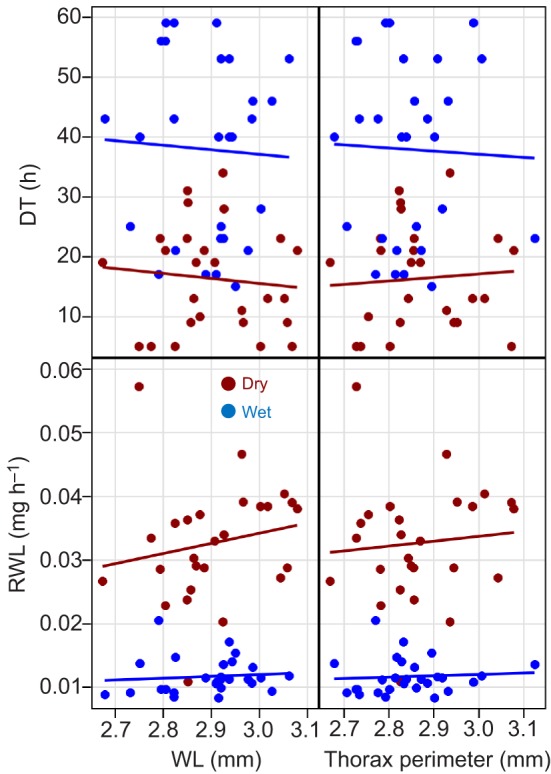
**Effects of body size on DT and RWL under the two treatments.** Body size was measured as mean wing length (WL) and thorax perimeter. Regression lines have been added to convey trends although the relationships are not statistically significant (see Results). For both WL and thorax size, sample sizes were *N*_dry_=26 and *N*_wet_=28.

**Fig. 4.**
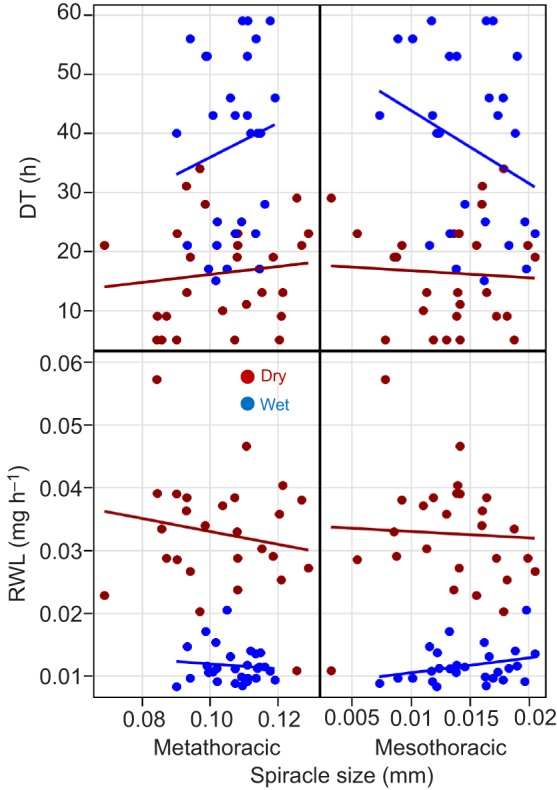
**Effects of spiracle size on DT and RWL under the two treatments.** Spiracle size was measured as the length of the mesothoracic and metathoracic spiracles. Regression lines have been added to convey trends although the relationships are not statistically significant (see Results). *N*_dry_=26 and *N*_wet_=28.

Contrary to expectation based on the ratio of surface area to volume, the effects of body size, typically measured by WL and thorax perimeter on DT and on RWL were not significant (*P*>0.21, *F*_1,51_<1.57; Fig. 3). The length, perimeter and area of both the mesothoracic and metathoracic spiracles were positively correlated with WL and thorax perimeter (0.11<*r*<0.53, not shown), but their effects on survival and RWL were all insignificant (*P*>0.13, *F*_1,51_<2.35, Fig. 4), regardless of whether body size index was included in the models.

Coefficient of variation values were substantially lower for spiracle length than for area (Table S1). Because thoracic spiracles open and close along their width (Movies 1 and 2), spiracle length is less sensitive to the degree to which it is opened. As the spiracle may have settled in a random state upon death, the degree of spiracle opening might add considerable variation to its area and perimeter. Therefore, we used spiracle length in subsequent analyses.

#### CHCs

**Fig. 5.**
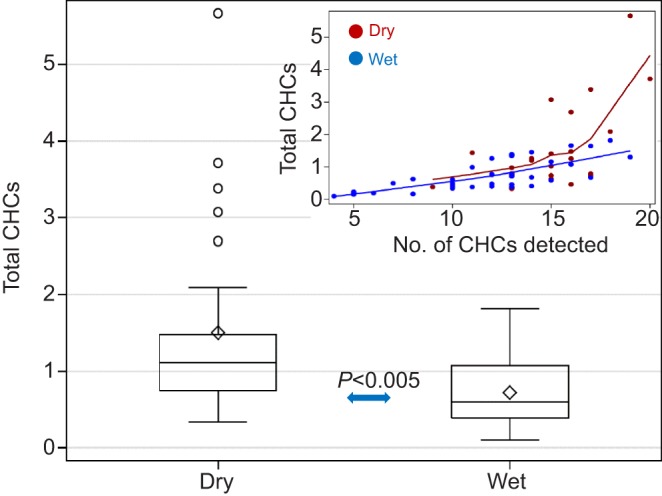
**Differences in total cuticular hydrocarbons (CHCs) between treatments.** The larger variance of the dry treatment (box plot and inset) was detected by Brown and Forsythe's test of homogeneity of variance (*P*<0.019). The difference between means was evaluated by Welch's ANOVA. Inset shows the relationship between total CHCs and the number of CHC peaks (note the 5 extreme values). *N*_dry_=26 and *N*_wet_=38.

A total of 30 CHCs were putatively identified in 64 females that were subjected to the laboratory desiccation assay. Surprisingly, the total amount of CHCs in the mosquitoes subjected to the dry treatment was higher than in those subjected to the humid treatment (*P*<0.006, Welch's ANOVA) and a larger variance was also evident in that group (*P*<0.019, Brown and Forsythe's test; Fig. 5). In part, this difference was produced by five mosquitoes (20%, *N*_dry_=26) that had considerably larger amounts of CHCs (Fig. 5), but even excluding these individuals, the total amount of CHCs was elevated in that group, suggesting that under harsh desiccation conditions, mosquitoes can respond by elevating the total amount of CHCs (Fig. 5). However, higher amounts of total CHCs (as well as body size-adjusted total CHCs) did not increase DT or decrease RWL (*P*>0.35 ANCOVA, not shown).

**Fig. 6.**
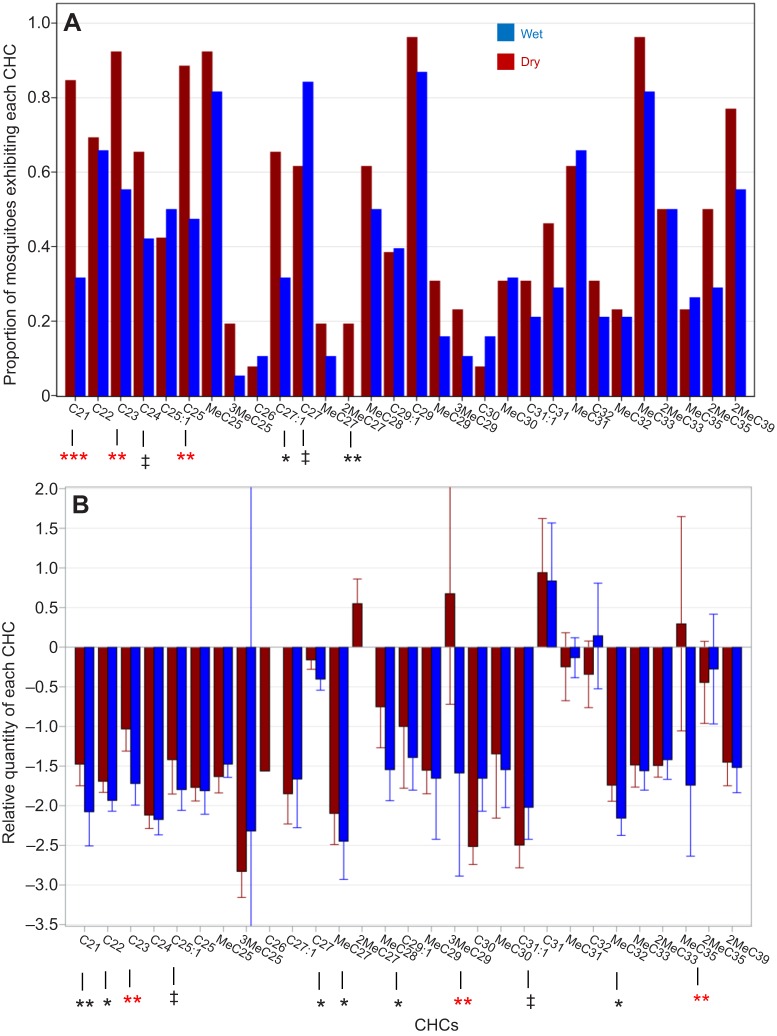
**Variation in CHC composition of G3 mosquitoes between treatments.** (A) The proportion of mosquitoes exhibiting each CHC as a measure of abundance. (B) The relative quantity of each CHC using log contrast-transformed values. Lines represent 95% confidence interval (CI) of the mean (single test level). Significant differences in frequency between treatments are indicated by asterisks below bars. Black and red asterisks indicate significance at the individual and multi-test level, respectively (^‡^*P*<0.1, *P<0.05, **P<0.01 and ***P<0.001).

Considerable variation was observed among individual females. Although a high proportion of mosquitoes exhibited *n*-C29, methyl-branched C33 (MebrC33) and MebrC25 (91%, 88% and 86%; Fig. 6), no CHC was detected in all of the G3 mosquitoes and only 13 of 30 CHCs were detected in 50% or higher proportion. The methyl-alkane 2MebrC27 was detected in 5 of 26 mosquitoes subjected to the dry treatment but in 0 of 38 from the humid treatment (*P*<0.008, Fisher exact test; *P*>0.05 at the multi-test level; Fig. 6). The alkanes *n*-C21, *n*-C23 and *n*-C25 were found in a higher proportion of the mosquitoes subjected to the dry treatment (*P*<0.01, Fisher exact test; Fig. 6), whereas C27:1 exhibited the reverse trend. The relative quantity of the CHCs, expressed as the log contrast-transformed values of CHCs (Fig. 6), showed differences between treatments in nine of 25 CHCs (overall *P*<0.0001, binomial test). Three (MebrC35, 3MebrC29 and *n-C*23) of the nine CHCs were significant in the multi-test level (Fig. 6).

To evaluate the effects of total CHCs as well as CHC composition on DT, we used a stepwise regression model (Materials and methods). A set of six CHCs and treatment accounted for 71% of the variation in DT (treatment alone accounted for 38%), and all were statistically significant (*P*<0.05; Table 2). The effects of *n*-C25 and *n*-C27 on DT were positive, whereas those of *n*-C24, *n*-C32, MebrC29 and MebrC31 were negative. Of these, *n*-C25, *n*-C27 and MebrC29 were elevated in the dry treatment (Fig. 6). Considering RWL, 73% of its variation was accounted for by the effect of the assay RH (treatment), 3MebrC29, MebrC29 and *n*-C21. In a model containing the relative abundance of the selected CHCs, increasing total CHC amount decreased RWL (Table 2). Variable-selection regression models may produce misleading results based on spurious correlations unless independent data are available for validation tests. Notably, MebrC29 is the only CHC that showed a negative effect on DT and a positive effect on RWL.

**Table 2.**
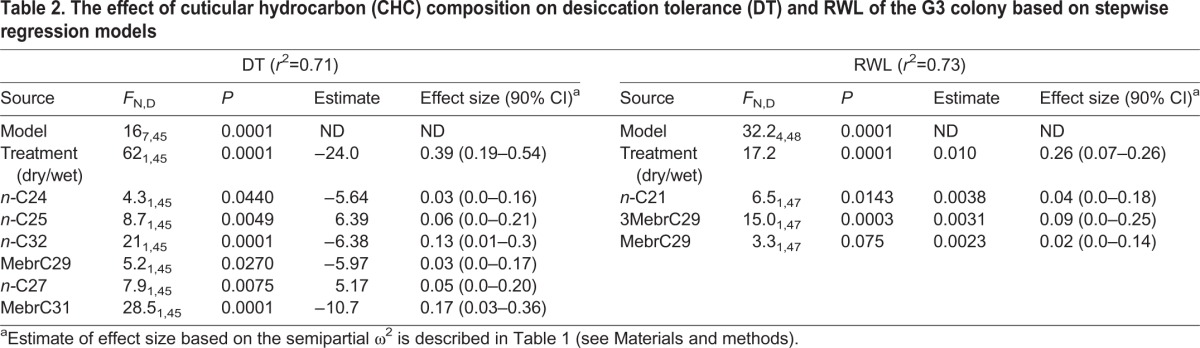
**The effect of cuticular hydrocarbon (CHC) composition on desiccation tolerance (DT) and RWL of the G3 colony based on stepwise regression models**

### Field experiment

**Fig. 7.**
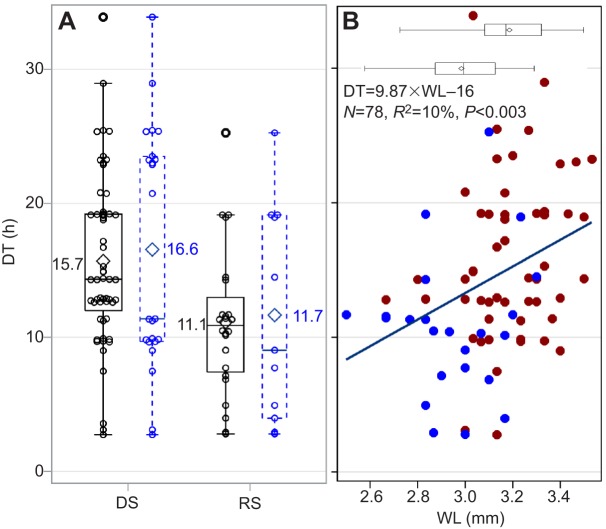
**Seasonal difference in DT in wild gravid *Anophelescoluzzii* and variation in body size in relation to DT.** (A) DT in the rainy season (RS) and dry season (DS). Black and blue distributions represent, respectively, the full number and a subset of specimens used in the desiccation assay and in the morphometric and CHC analyses. The seasonal difference is highly significant over the total data (*P*<0.0003, *N*=81, Wilcoxon test one-way) and significant in the subset (*P*<0.047, *N*=34, Wilcoxon test one-way). (B) Seasonal variation in body size (measured as WL) is summarized in the horizontal box whisker (top; see Results). Red and blue dots represent DT values of gravid mosquitoes from the DS and RS, respectively. The regression line shows the relationship of DT and WL regardless of season (see Results).

A total of 81 gravid *A. coluzzii* mosquitoes were subjected to the desiccation assay in the field but only 34 intact specimens were available for morphometric and CHC analyses. They represented 23 and 11 gravid females collected during the DS and the RS, respectively. In agreement with predictions (Introduction), the mean DT of females collected in the DS (15.7) was higher than that of females collected in the RS (11.1, *P*<0.0003, *N*=81, one-way Wilcoxon test; Fig. 7A). In the subset of mosquitoes used in the morphometric and CHC analyses, however, the difference was only marginally significant (*P*<0.093, *N*=34, one-way Wilcoxon test; Fig. 7A), probably reflecting the smaller sample size.

#### Body size and spiracle size effects on DT

Body size measured by WL in the (early) DS was larger than that in the RS (3.18 versus 2.95 mm, *P*<0.001, *F*_1,76_=24.9; Fig. 7B), consistent with earlier findings (Huestis et al., 2012). The effect of WL on DT was significant overall (Fig. 7B); however, it was confounded by the effect of season because when season was included in the model, the body size effect was insignificant (*P*>0.1, ANCOVA), yet season remained significant (*P*<0.029, in the presence of body size). The interaction between season and WL was not significant (*P*>0.4, ANCOVA).

Spiracle size (length) of both the metathoracic and mesothoracic spiracles was correlated with thorax size (*r*=0.44 and 0.39, respectively, *P*<0.014) more than with WL (0.24 and 0.13, respectively, *P*>0.12). Indeed, thorax size was modestly correlated with WL (*r*=0.62, *P*<0.001, *N*=38). Metathoracic spiracle size and mesothoracic spiracle size were positively correlated with each other (*r*=0.51, *P*<0.0007).

**Fig. 8.**
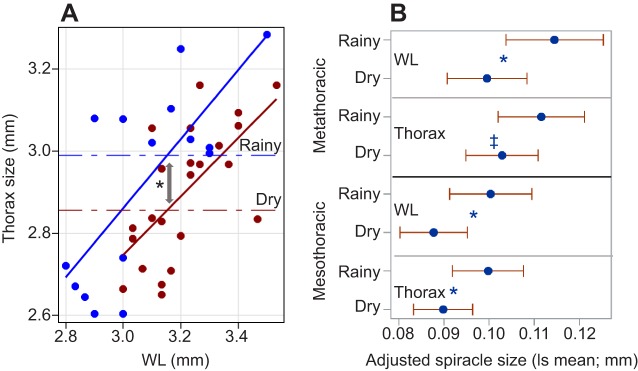
**Seasonal allometry in thorax–wing size and spiracle size in relation to both wing and thorax size.** (A) Variation in thorax size in relation to WL by season (red and blue denote DS and RS, respectively). The effect of season (*P*<0.012) and WL (*P*<0.001) in a covariance analysis (interaction term was not significant) are shown. Horizontal lines denote mean thorax size of each season adjusted to WL values (least square means). (B) Mean spiracle size adjusted to WL and thorax size and their 95% CI computed in a covariance analysis. The significance of one-way tests of the hypothesis of smaller relative spiracle size during the DS is shown in blue (*P<0.05 and ^‡^0.05<*P*<0.1).

Seasonal allometry was found between WL and thorax size (Fig. 8A), showing that for the same WL, expected thorax size in the DS (least square means =2.856 mm) was smaller than that in the RS (least square means=2.989 mm, *P*<0.013, ANCOVA; Fig. 8A). This difference (0.133 mm) amounts to 70% of the standard deviation of thorax size (0.18 mm; Table S1). The hypothesis of relatively smaller spiracle size during the DS was tested using least square means in ANCOVA (one-side test). The interactions between season and both measures of body size were insignificant and, therefore, were removed. The hypothesis was confirmed for both spiracles (*P*<0.05), except for the metathoracic spiracle with respect to thorax size (*P*<0.084; Fig. 8B), indicating that during the DS, spiracle size is smaller than expected from body size even relative to the already smaller thorax size during the DS.

**Fig. 9.**
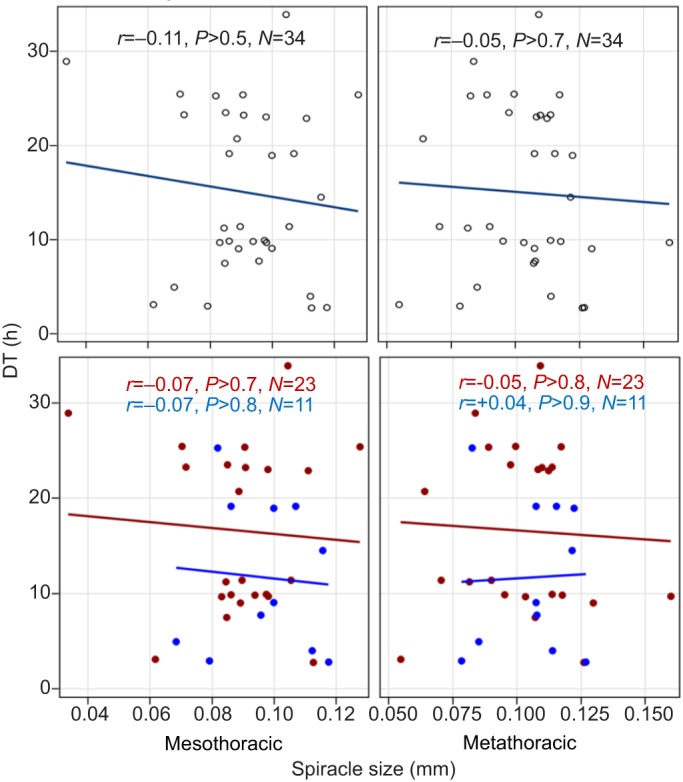
**Effects of mesothoracic and metathoracic spiracle size (length) on DT.** Top, overall results; bottom, results by season. Red and blue denote DS and RS, respectively. Correlation coefficients and their significance are shown for the top panels.

The effect of spiracle size on DT was evaluated in univariate and multivariate ANCOVA. Although a larger spiracle appeared to decrease DT, according to the hypothesis (Fig. 9), these effects were non-significant (including in multivariate analyses, not shown).

#### CHCs

During the DS, total CHC appeared elevated and its distribution wider than that in the RS, yet these differences were not significant (*P*>0.15, Welch's ANOVA, *F*_1,33_=1.69). Total CHCs did not vary significantly in relation to body size by season or across seasons (*P*>0.2). Finally, a higher amount of total CHCs appeared to increase DT in the DS but this effect was non-significant (*P*>0.3, ANCOVA, *F*_1,30_=0.8), after removal of non-significant interactions, as was also the case during the RS (*P*>0.3 ANCOVA, *F*_1,30_=0.81).

**Fig. 10.**
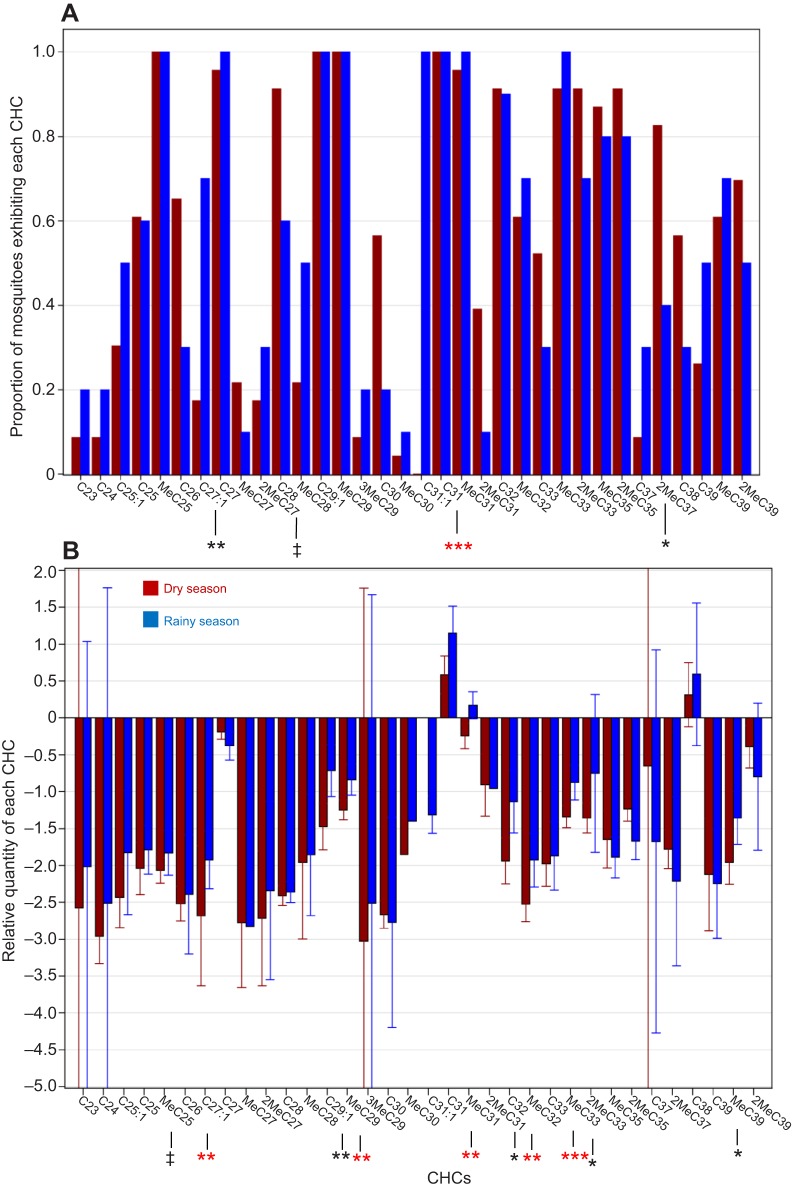
**Seasonal variation in CHC composition of *A. coluzzii*.** (A) The proportion of mosquitoes exhibiting each CHC as a measure of its abundance. (B) The relative quantity of each CHC using log contrast-transformed values. Significant differences in frequency between seasons are indicated by asterisks below bars. Black and red asterisks indicate significance at the individual and multi-test level, respectively (^‡^*P*<0.1, *P<0.05, **P<0.01 and ***P<0.001). *N*_dry_=23 and *N*_rainy_=12.

The composition of CHCs was overall similar across seasons (Fig. 10A). Only C31:1 was season specific; it was present in all the RS specimens (*N*=10) but in none of the DS specimens (*N*=23, *P*<0.001, Fisher exact test; Fig. 10A). Similarly, the alkene C27:1 was present in a smaller fraction of DS mosquitoes (*P*<0.006, Fisher exact test; Fig. 10A), whereas 2MebrC37 and *n*-C28 were detected in higher proportions of DS mosquitoes (*P*<0.035, and *P*<0.054, respectively, Fisher exact test), suggesting these CHCs were more concentrated during the DS. Considering the log contrast-transformed values (Fig. 10B), significant seasonal differences in relative quantity were detected in nine of 21 CHCs, indicating a seasonal shift in composition (*P*<0.0001, binomial test). Further, six of the nine CHCs were also significant in the multi-test level (Fig. 10B).

To evaluate the effects of total CHCs as well as CHC composition on DT, we used a stepwise regression model (including season). A set of five CHCs and total CHCs accounted for 66% of the variation in DT and all were statistically significant (*P*<0.05; Table 3). The effects of the total CHCs, *n*-C28, *n*-C33, and MebrC33 were positive, whereas those of C29:1 and *n*-C31 were negative. Of these, MebrC33 and C29:1 were significantly different between seasons (Fig. 10), although MebrC33 was reduced during DS, contrary to its positive effect on DT, while C29:1 was reduced during DS, in accordance with its negative effect on DT. Likewise, n-C28 was more common in the DS, in accordance with its positive effect on DT.

**Table 3.**
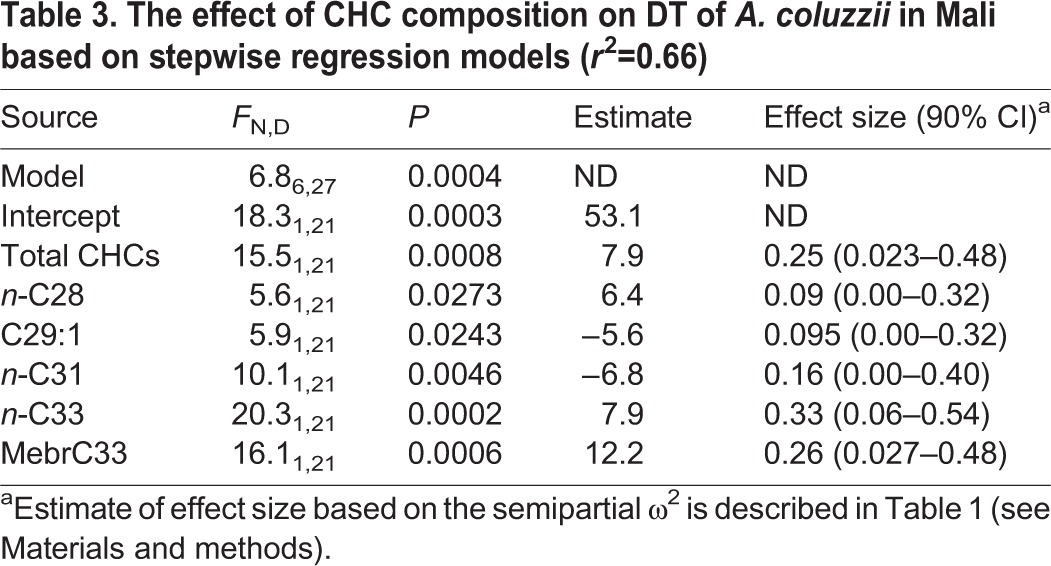
**The effect of CHC composition on DT of *A. coluzzii* in Mali based on stepwise regression models (*r*^2^=0.66)**

## DISCUSSION

Using laboratory and field experiments, we have evaluated the effects of spiracle size, CHC composition and total CHC amount on desiccation tolerance of the African malaria mosquito. Many studies have speculated on the importance of tolerance to aridity for the persistence of members of the *A. gambiae* complex in dry environments (Bayoh et al., 2001; Coluzzi et al., 1985, 1979; Huestis and Lehmann, 2014; Lehmann and Diabate, 2008) and about the effects of the spiracles and CHCs in minimizing water loss (Benoit and Denlinger, 2007, 2010; Fouet et al., 2012; Gray and Bradley, 2005; Gray et al., 2009; Hidalgo et al., 2014; Huestis et al., 2012; Mamai et al., 2014; Nagpal et al., 2003; Wagoner et al., 2014). Our results have produced support for at least three of our five hypotheses (see Introduction): during the DS, *A. coluzzii* mosquitoes exhibited higher DT, smaller spiracle size relative to body size and specific seasonal changes in their CHC composition, some of which correlated with increased DT. These findings, and especially the seasonal allometries in thorax and spiracle size of *A. coluzzii*, are consistent with local persistence throughout the DS, referred to as aestivation (Adamou et al., 2011; Dao et al., 2014; Lehmann et al., 2010). However, there was insufficient evidence that smaller spiracle size increased DT, possibly because of limited sample size. There was partial congruence between the results of the field and the laboratory experiments in respect to the roles of these mechanisms on DT.

### Effects of body and spiracle size on DT

Consistent with our results, a seasonal increase in WL during the early DS was reported based on specimens collected a year earlier (2010) in Thierola (Huestis et al., 2012). As predicted based on surface-to-volume ratio (Gray and Bradley, 2005), larger WL increased DT in a univariate regression (Fig. 7); however, this effect did not persist within season and was confounded with season. In the laboratory experiment, no effect of body size (WL and thorax perimeter) on DT or RWL was detected (Fig. 3). Possibly, the G3 mosquitoes used were less variable in size than wild mosquitoes as suggested by their coefficient of variation (Table S1).

Relative to body size (WL), the thorax of *A. coluzzii* was smaller in the DS than in the RS (Fig. 8). Moreover, during the DS, the thoracic spiracles relative to WL and relative to thorax perimeter were smaller than they were during the RS (Fig. 8). Smaller spiracle size (relative to body size) was concurrent with elevated DT during the DS (although the correlation was not statistically significant), in agreement with it being a mechanism to minimize transpiration. It may also be explained by the hypothetically lower demands for gas exchange due to restricted movement of aestivating mosquitoes (Huestis et al., 2012). The relative reduction in thorax size is consistent with this explanation and further suggests that during the DS, mosquitoes allocate fewer resources to locomotion, yet the larger wings suggest that mosquitoes carry heavier loads (blood, sugar meals or water) during their short foraging excursions. Such allometries highlight that, as in other diapause developmental programs, the insect responds to certain token stimuli before metamorphosis (Denlinger, 2002; Denlinger and Armbruster, 2014; Huestis and Lehmann, 2014), consistent with aestivation. Could the shift in morphology be a result of differential survival during the DS instead? Accordingly, the distribution of the spiracular ratios during the RS would subsume that of the DS. However, contrary to this prediction, over 75% of spiracle:wing ratio values during the DS were below the lowest 95% confidence interval of the corresponding RS distributions (Fig. 8). Further study into the seasonal and spatial variation in this morphometric signal will help determine its potential as a marker of aestivation as well as its function.

The ‘spiracular index’ (ratio of the length of the mesothoracic spiracle to the length of the thorax) was compared among African anophelines with respect to the aridity of their habitat (Vinogradskaia and Detinova, 1978). The members of *A. gambiae s.l.* were grouped based on ‘intermediate index’ as moderately xero-resistant although large differences were found between mosquitoes from savanna versus forest habitats. Seasonal variation in spiracle size was suggested in *A. stephensi* from arid regions in India, showing a smaller spiracle during the DS (Nagpal et al., 2003). Wagoner and colleagues (2014) have examined the variation in spiracle size between G3 mosquitoes raised from eggs under short and long photoperiod regimes. They found that, contrary to their hypothesis, mosquitoes exposed to short photoperiod, corresponding to the DS, exhibited larger WL and larger relative mesothoracic spiracle length than mosquitoes exposed to long photoperiod. Direct evaluation failed to detect an effect of spiracle size on DT in *A. coluzzii* and in the G3 colony of *A. gambiae* (Fig. 4). Altogether, we conclude that variation in spiracle size in *A. coluzzii* may be a secondary mechanism underlying its DT. Nonetheless, transpiration through the spiracles might be regulated behaviorally by closing the spiracles (Gray and Bradley, 2006; Hadley, 1994; Krafsur, 1971), as shown in Movies 1 and 2 morphologically by the setae (cuticular ‘hairs’) that surround the opening (Mamai et al., 2016).

### Effects of the total amount and composition of CHCs on DT

During the DS, the total amount of CHCs of *A. coluzzii* appeared higher than that during the RS, especially in a subset of the DS mosquitoes, although the difference was not significant (Fig. S3). Moreover, higher total CHCs alone did not increase DT in *A. coluzzii* and in G3 mosquitoes. Likewise, total CHCs alone did not reduce RWL of G3 mosquitoes. Conversely, in combination with particular CHCs, the total amount of CHCs elevated DT in *A. coluzzii*, suggesting total CHC amount plays a modest role as a mechanism conferring elevated DT on members of the *A. gambiae* complex.

Adaptation to desiccation stress during two independent selection experiments on *Drosophila* showed stability in the total amount of CHCs, but the relative abundance of longer chain CHCs increased, reducing RWL (Gibbs et al., 1997; Kwan and Rundle, 2010). Consistent with our results, G3 mosquitoes exposed to short photoperiod and low RH, simulating the DS, exhibited greater total CHCs than mosquitoes exposed to long photoperiod and high RH, and also revealed differences in CHC composition between these groups (Wagoner et al., 2014). Seasonal changes in the CHC composition were detected in *A. coluzzii* (Fig. 10A), with the notable absence of C31:1 from all DS specimens as opposed to its presence in all RS specimens. The alkene C27:1 was detected in a smaller fraction of DS mosquitoes, whereas 2MebrC37 and *n*-C28 were detected in higher proportions of DS mosquitoes. Similarly, the relative quantity of C27:1, MebrC29, MebrC31, MebrC32 and MebrC33 was reduced in the DS (Fig. 10B). The reductions in the relative abundance of alkenes and methylated alkanes followed expectations based on studies showing that adding a double bond or a methyl group to an alkane (of a given length) reduces its melting temperature by ∼10–50°C, depending on the position where the change is made (Gibbs and Pomonis, 1995; Stinziano et al., 2015). According to the phase shift model, desiccation-resistant insects, especially in hot and arid environments, possess a layer of CHCs that melt at higher temperatures. Thus, the elimination of the ubiquitous RS alkene C31:1 and the reduction in the relative quantity of C27:1 as well as the methyl-branched alkanes from *A. coluzzii* during the DS fit well with this model. In evaluating direct effects on DT, a more complex pattern arises, possibly demonstrating the interactive nature of CHCs in forming the permeability of the epicuticle (Gibbs and Rajpurohit, 2010). Thus, both ‘expected’ reductions in the relative quantity of the alkene C29:1 and elevated amounts of *n*-C28 and C33 were observed together with the ‘unexpected’ reduction of C31 and the increase in MebrC33 (Table 3). Despite growing knowledge on lipid-phase transitions, how different compounds actually interact to determine cuticular impermeability to water is poorly understood, and likely includes interactions among CHCs, other lipids, proteins and probably melanization (Gibbs and Rajpurohit, 2010).

Several CHCs showed consistent patterns, increasing during the DS and having a positive effect on DT (*n*-C28) or decreasing during the DS and having a negative effect on DT (C29:1), yet disparities were also observed, such as the reduction of MebrC33 during the DS and its positive effect on DT. Notably, not all the CHCs that were included in the seasonal comparisons were included in the analysis of the effects of the CHCs on DT (Materials and methods). The laboratory experiment reinforced that decreasing MebrC29 enhanced the DT of G3 mosquitoes (Table 2), consistent with its lower amount in *A. coluzzii* during the DS (above). Similarly, decreasing MebrC31 increased DT of the G3 mosquitoes (although it showed no effect on RWL) and was found in a lower amount during the DS in *A. coluzzii* (above). The remaining four CHCs that affected DT and the two affecting RWL in the laboratory experiment, however, showed no effect on *A. coluzzii*. The differences in CHC composition between the laboratory and the field experiments (Table 4) may be attributed to the gonotrophic state (unfed and gravid, respectively), the genetic makeup of the G3 colony (a hybrid between *A. coluzzii* and *A. gambiae* from locations distant to our field site), and/or the conditions they have been maintained under. These results highlight the uncertainty in generalizing patterns from the laboratory to the field and the need for validating studies. Yet, concordance between the field and laboratory results substantiate the role of CHC composition and total CHC amount as underlying mechanisms conferring DT in anopheline mosquitoes.

**Table 4.**
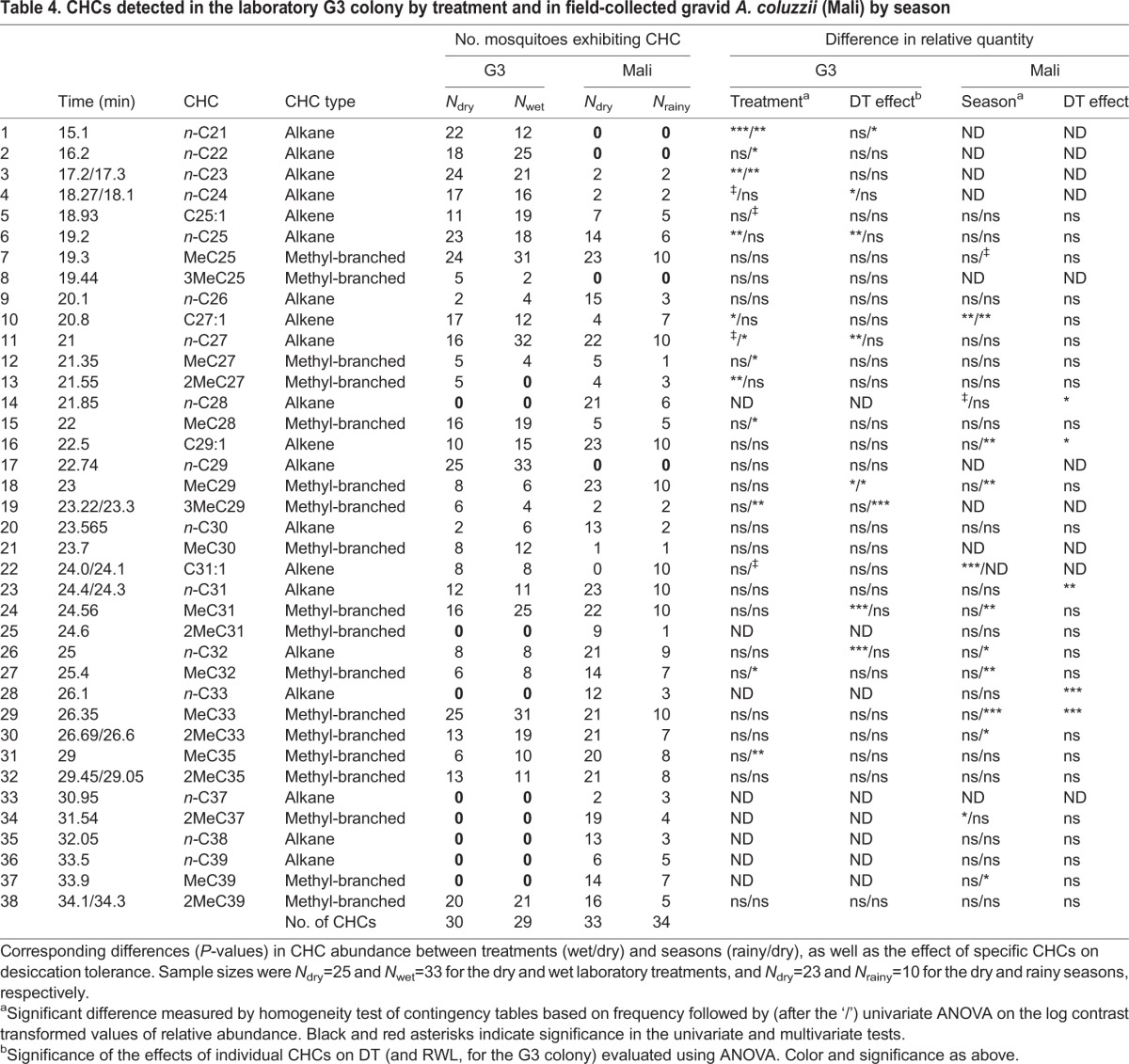
**CHCs detected in the laboratory G3 colony by treatment and in field-collected gravid *A. coluzzii* (Mali) by season**

The evidence that variation in CHC composition mediates DT in *A. coluzzii* is a prerequisite for serving as a ‘dual-role trait’ that might lead to ecological speciation if it also affects mate recognition (Chung and Carroll, 2015). Caputo and colleagues (2007) compared CHC composition of female *A. coluzzii*, *A. gambiae* and *A. arabiensis* from three villages in Burkina Faso and found differences mostly within taxa between villages rather than between taxa, suggesting environmental constraints shaping variation in CHC composition. Across villages, 2MebrC35 showed a degree of differentiation between *A. arabiensis* and *A. gambiae*, whereas MebrC29 showed a degree of differentiation between *A. coluzzii* and *A. gambiae*. All mosquitoes used in their analyses were collected during the RS (October), precluding seasonal comparison. The qualitative absence of C29:1 from *A. coluzzii* in the DS shown in our study (above) stands out as a markedly large seasonal effect on CHCs. We found no variation in 2MebrC35, yet MebrC29, which was higher in *A. gambiae* and *A. arabiensis* (Caputo et al., 2007), was elevated during the RS (Table 4 and Fig. 10), consistent with *A. coluzzii* being the most arid-tolerant in that region.

The elevated total CHCs and change in CHC composition in the mosquitoes exposed to dry versus humid treatments suggest a hardening response as described in several *Drosophila* species and differ from findings during acclimation (Stinziano et al., 2015). Within hours after recovery from high desiccation stress, *Drosophila melanogaster* females exhibit changes in CHC profile (without increasing the total CHC quantity) that increase DT by decreasing RWL by ∼30% (Bazinet et al., 2010; Kalra et al., 2014). In our assay, mosquitoes were not allowed to recover, so the increased total CHCs (Fig. 5) may represent the initiation of the hardening response.

### DT of malaria mosquitoes and persistence in arid environments

In the G3 mosquitoes, the ambient RH, RWL, BWA and, to a lesser degree, BWCD accounted for 82% of the variation in DT. Notably, these results suggest variability in mosquito capacity to withstand the minimal concentration of water in the body in addition to RWL, whereas hydration at the onset of the assay was the only determinant detected by a previous study comparing DT in *A. arabiensis* and *A. gambiae* (Gray and Bradley, 2005). Our estimate of the effect size of BWCD (including its interaction with RWL) was less than a third of that of RWL (estimated by partitioning *R*^2^ among all factors of the model, not shown). The significant effect of wet mass on DT, as opposed to that of end mass and dry mass, and its high correlation with RWL suggest that hydration at the onset of the desiccation assay was the primary factor determining the mosquito DT (aside from the assay RH). As the hydration level of a laboratory mosquito reflects merely the time and size of the last sugar meal(s) it took prior to the assay, rather than an inherent difference in body water content, we conclude that the G3 colony maintains low innate variation in DT, possibly explaining why variation in spiracle size had negligible effect on it.

During the 7 month-long DS in the Sahel, when RH seldom rises above 25%, survival for small terrestrial insects must pose an enormous challenge (Huestis and Lehmann, 2014). Because the field desiccation assay was performed at <10% RH, as opposed to ∼25% RH of the laboratory assay, we cannot compare the DT of *A. coluzzii* and the G3 colony, yet the difference is probably not measured in orders of magnitude (Table S1). The higher DT of *A. coluzzii* during the DS may be considered as evidence for its importance during aestivation. However, if this elevated DT is a primary component of aestivation, could it be manifested by a mere 40% difference (rather than, say, 400%)? How many days longer would a female *A. coluzzii* survive in the midst of the DS compared with her granddaughter during the RS if this was the primary mechanism of her aridity tolerance? Notably, there was considerable overlap in the seasonal distributions of DT despite being different statistically (Fig. 7). A similar magnitude of difference in DT was recorded between various species and populations of *A. gambiae s.l.* (Gray and Bradley, 2005; Gray et al., 2009; Lee et al., 2009), which was presumably smaller than anticipated. Even in hibernating *Culex pipiens*, reduction of RWL in diapausing females amounted to merely 46% of that of non-diapausing females (Benoit and Denlinger, 2007). Based on these considerations, we suspect that, unless higher DT is manifested only by aestivating mosquitoes while in hidden shelters (which are yet to be found), the physiologically elevated DT as measured here in *A. coluzzii* contributes less to its persistence throughout the 7 month-long DS than its behavior (Huestis and Lehmann, 2014). Unlike hibernating insects that withstand the winter mostly immobilized by near-freezing temperatures, *A. coluzzii* can move around and replenish its body water content from blood meals, sugar sources and water in or near wells or water pots in houses (although these water sources are not suitable for reproduction). Their hitherto unknown refugia might provide elevated RH and optimal temperatures (Huestis and Lehmann, 2014; Kessler and Guerin, 2008). Despite the growing understanding of the mechanisms allowing their presence in arid conditions, integrating the physiological and behavioral adaptations of these species to an empirically demonstrated strategy remains to be achieved.
